# Theta Neurofeedback Training Supports Motor Performance and Flow Experience

**DOI:** 10.1007/s41465-021-00236-1

**Published:** 2021-12-22

**Authors:** Kathrin C. J. Eschmann, Lisa Riedel, Axel Mecklinger

**Affiliations:** 1 Experimental Neuropsychology Unit, Department of Psychology, Saarland University, Saarbrücken, Germany; 2 Cardiff University Brain Research Imaging Centre (CUBRIC), School of Psychology, Cardiff University, Cardiff, UK; 3 Faculty of Sport Science, Leipzig University, Leipzig, Germany

**Keywords:** Theta oscillations, Neurofeedback, Training, Flow, Motor performance, Sports

## Abstract

Flow is defined as a cognitive state that is associated with a feeling of automatic and effortless control, enabling peak performance in highly challenging situations. In sports, flow can be enhanced by mindfulness training, which has been associated with frontal theta activity (4-8 Hz). Moreover, frontal-midline theta oscillations were shown to subserve control processes in a large variety of cognitive tasks. Based on previous theta neurofeedback training studies, which revealed that one training session is sufficient to enhance motor performance, the present study investigated whether one 30-minute session of frontal-midline theta neurofeedback training (1) enhances flow experience additionally to motor performance in a finger tapping task, and (2) transfers to cognitive control processes in an *n*-back task. Participants, who were able to successfully upregulate their theta activity during neurofeedback training (responders), showed better motor performance and flow experience after training than participants, who did not enhance their theta activity (non-responders). Across all participants, increase of theta activity during training was associated with motor performance enhancement from pretest to posttest irrespective of pre-training performance. Interestingly, theta training gains were also linked to the increase of flow experience, even when corresponding increases in motor performance were controlled for. Results for the *n*-back task were not significant. Even though these findings are mainly correlational in nature and additional flow-promoting influences need to be investigated, the present findings suggest that frontal-midline theta neurofeedback training is a promising tool to support flow experience with additional relevance for performance enhancement.

## Introduction

Experience of a flow state is highly desirable in a large variety of situations, in which peak performance is required. First defined by Csíkszentmihályi (1975, 1990), flow describes a highly functional state that is associated with a feeling of automatic and effortless control while being completely absorbed and focused on the activity at hand. The subjective flow experience is inevitably connected to active performance in a challenging task. For flow to occur, both skill level of the person and challenge level of the situation need to be high but balanced, leading to neither boredom nor overload (Csíkszentmihályi, 1997). Consequently, flow proves to be beneficial when experts act in challenging situations. Support comes from studies showing that flow experience predicts cognitive (e.g., Engeser & Rheinberg, 2008) and athletic performance outcomes (e.g., Jackson et al., 2001; Koehn et al., 2013). However, although athletes reported to be able to control flow frequency and quality at least to some extent, flow states seem to occur very rarely, leaving accessible performance potential being unused (Swann et al., 2012). Moreover, individual differences in the proneness to experience flow exist and have been associated with dopamine receptor availability in the dorsal striatum, a region important for reward processing and intrinsic motivation (de Manzano et al., 2013).

In sports, training interventions applying mindfulness meditation have been used to improve flow experience (Gardner & Moore, 2012; Kaufman et al., 2009). Originally stemming from Buddhist meditation traditions, mindfulness meditation is defined as a non-judgmental state of attention to experiences happening in the present moment (Baer, 2003; Hölzel et al., 2011). Many studies were able to demonstrate positive effects of meditation-based mindfulness interventions on motor performance and flow experience not only in athletes but also in novices, who learned new movements (e.g., Aherne et al., 2011; Scott-Hamilton et al., 2016; Zhang et al., 2016). Mindfulness meditation is assumed to support self-regulation by increasing attentional and cognitive control processes (Tang et al., 2015; Teper et al., 2013). Following meditation practice, participants showed enhanced conflict monitoring and emotion regulation reflected in improved attentional performance (e.g., Chiesa et al., 2011; Slagter et al., 2007; Tang et al., 2007) and reduced emotional reactions (e.g., Chambers et al., 2008; Ortner et al., 2007; Robins et al., 2012). Neuroimaging studies supported these findings by revealing greater mindfulness-driven activation in the anterior cingulate cortex (ACC) and the prefrontal cortex (PFC), two brain regions important for conflict detection and monitoring as well as execution of top-down control (Allen et al., 2012; Hölzel et al., 2007; Lutz et al., 2013; Tang et al., 2010). It is assumed that mindfulness meditation increases the sensitivity to affective cues that signal the need for cognitive control processes, which in turn downregulate limbic brain regions that are important for emotion processing, allowing flow experience to occur (Hölzel et al., 2011; Tang et al., 2015; Teper et al., 2013). Studies investigating the neurophysiological underpinnings of flow experience support this assumption by revealing increased lateral PFC activation and decreased amygdala activation during flow experience in challenging task situations as compared to boredom or overload (Ulrich et al., 2014, 2016; Yoshida et al., 2014). These findings were interpreted to reflect enhanced cognitive control processes, such as task goal maintenance and top-down control, and reduced negative arousal, respectively. In addition, flow experiences were also shown to require an upregulation of and increased functional connectivity between brain areas involved in cognitive control and reward processing (Huskey et al., 2018a, 2018b; Klasen et al., 2012). More specifically, in situations of high flow experience, the highly rewarding nature of flow increases reward-related processing, which then modulates the deployment of cognitive control. Interestingly, flow experience was not only shown to require downregulation of limbic brain areas but also reduced activation of brain areas involved in self-referential processing, such as the ACC and medial PFC (Klasen et al., 2012; Ulrich et al., 2014, 2016, 2018). Additionally to increased reward-modulated cognitive control processes, flow experience might thus be associated with reduced signaling of the need for cognitive control due to better maintenance of clear task goals and lowered self-referential processes (Harris et al., 2017b; Ulrich et al., 2014).

Numerous studies have shown that cognitive control processes are accompanied by frontal-midline (FM) theta oscillations (4-8 Hz) that scale with task difficulty (e.g., Cavanagh & Frank, 2014; Cooper et al., 2017; Eschmann et al., 2018). For instance, FM theta activity was shown to be elevated in tasks with high cognitive load (e.g., Griesmayr et al., 2010; Jensen & Tesche, 2002; Roberts et al., 2014) and to decrease with advancing interference resolution (e.g., Ferreira et al., 2014; Spitzer et al., 2009; Waldhauser et al., 2014). Specifically, theta activity decreased from the first to the second half of a retrieval practice motor memory task, indicating that theta activity is an indicator of concurrent motor memory interference (Tempel et al., 2020). Consequently, FM theta activity has been proposed to reflect the need for cognitive control that subsequently gets implemented via theta phase coherence between theta source regions and other task-relevant brain areas (Cavanagh & Frank, 2014). FM theta oscillations were suggested to originate from brain areas that also play a role in mindfulness meditation and flow experience, namely the ACC and PFC (Hsieh & Ranganath, 2014), supporting the claim that cognitive control processes promote the flow-enhancing benefits of mindfulness meditation. Furthermore, heightened frontal theta activity has been linked to meditation practices, deepness of meditative states (Cahn & Polich, 2006; DeLosAngeles et al., 2016; Lomas et al., 2015; Takahashi et al., 2005), and importantly also to the subjective experience of flow (Katahira et al., 2018). Based on the overlapping neurophysiological characteristics of cognitive control processes, mindfulness meditation, and flow experience, the question arises whether flow experience can be enhanced not only by meditation interventions but also by directly modulating FM theta activity.

Electrophysiological signals, such as the amplitude of a certain frequency band, can be up- or downregulated with the method of neurofeedback training (NFT). Feedback provided via a closed-loop brain computer interface (BCI) thereby allows neurofeedback users to learn the self-regulation of their own brain activity (e.g., Gruzelier, 2014b; Huster et al., 2014). Previous studies demonstrated that one session of theta NFT is sufficient to improve motor performance in a finger tapping task, suggesting facilitated consolidation of motor memory as a function of training (Reiner et al., 2014, 2018; Rozengurt et al., 2016). In a similar vein, FM theta NFT studies revealed training transfer to attention, cognitive control, and memory control processes (Enriquez-Geppert et al., 2014a; Eschmann & Mecklinger, 2021; Eschmann et al., 2020; Wang & Hsieh, 2013). Notably, transfer was evident for proactive rather than reactive control processes (Enriquez-Geppert et al., 2014a; Eschmann & Mecklinger, 2021), indicating that upregulation of FM theta activity especially supports active maintenance of task-relevant information and sensory biasing before cognitively demanding events (Braver, 2012). However, in these studies, seven or more training sessions were conducted, leaving the question unanswered whether one NFT session can enhance FM theta sufficiently to allow transfer to cognitive control processes and mental states, such as flow experience. Additionally, thus far, only one NFT study, in which actors trained to upregulate their sensory motor rhythm (12-15 Hz), assessed flow experience as a subjective measure of acting performance after training (Gruzelier et al., 2010). Given the single measurement after training, this study does not allow for any conclusions about the presence of training-induced changes of flow experience and their independence from objectively measured motor enhancement.

Given that flow experience occurs when skilled persons engage in challenging situations and thus it is inevitably connected to the task at hand, the present study assessed flow experience in a finger tapping task that was previously used to investigate transfer of FM theta NFT to motor performance (Rozengurt et al., 2016). The finger tapping task is thought to induce flow experience because it involves the execution of a newly but quickly learned motor movement, allowing for high ability of task execution, and the request to execute the movement as often and as accurately as possible, introducing high task difficulty. More specifically, we investigated whether a 30-minute FM theta NFT session (1) enhances flow experience during active performance of a challenging motor task and (2) leads to transfer to cognitive control processes in a demanding visual *n*-back task. Flow experience and task performances were assessed in a pre-post design with measurements directly before and after NFT and a follow-up measurement 24 hours thereafter. A previous FM theta NFT study that investigated enhancement of motor performance provided different strategies of brain activity modulation for the training and control group (Rozengurt et al., 2016). Consequently, training and transfer effects could not solely be attributed to FM theta upregulation. In order to rule out the influence of divergent training experiences, all participants in the present study trained to upregulate their FM theta activity during NFT and were provided with the same strategies for brain activity regulation. For further analyses, participants were split into responders and non-responders on the basis of their individual training success as it was done in other NFT studies (e.g., Autenrieth et al., 2020; Hanslmayr et al., 2005). This procedure allowed to compare both groups in training-induced performance outcomes and to investigate the influence of NFT success on transfer effects across all participants. Even though groups of responders and non-responders are compared, it should be noted that the present study has a mainly correlational nature. Therefore, all differences in flow experience and motor performance between responders and non-responders cannot be solely attributed to NFT but might equally likely be caused by a better ability of responders to perform in both the neurofeedback and transfer tasks. It was expected that participants, who successfully upregulated their FM theta activity, show better motor performance and enhanced flow experience directly after training and one day later compared to participants, who were less able to modulate their brain activity. If one NFT session is sufficient to improve cognitive control processes, faster reaction times and higher accuracy should also be obtained in the *n*-back task. Furthermore, the extent of FM theta upregulation during NFT was expected to scale with enhancement of motor performance, flow experience, and cognitive control from pretest to posttest but not from posttest to follow-up measurement. These differential effects were expected because training-induced changes should become apparent directly after training, whereas later changes should be mainly influenced by training-unrelated processes happening between posttest and follow-up measurement.

## Materials and Methods

### Participants

Overall, 48 German volunteers who were recruited from Saarland University’s student community (18 male, *M*_age_ = 22.44, range = 18-30 years) participated in the present study. One additional participant was excluded from statistical analyses due to wrong execution of the finger tapping task. Given that the relationship between neurofeedback success and training transfer was of main interest, a total sample size of *N* = 46 to detect a correlation of 0.40 with a power of 80% was determined with G*Power (Faul et al., 2007). Three additional participants were recruited in order to account for possible dropout due to behavioral or technical issues. Based on their NFT success, participants were classified into 25 responders (seven male, *M*_age_ = 22.24, range = 19-28 years) and 23 non-responders (eleven male, *M*_age_ = 22.65, range = 18-30 years). Participants were recruited based on the following characteristics that were determined via an online questionnaire prior to their visit to the laboratory. None of the recruited participants was an expert typist or musician. According to self-report, participants were healthy, had normal or corrected-to-normal vision, and no history of neurological or psychological disorders. Moreover, all participants were right-handed as indicated by the Edinburgh Handedness Inventory (Oldfield, 1971). Testing times were scheduled in accordance with each participant’s chronotype based on the German version of the Morningness-Eveningness Questionnaire (D-MEQ; Griefahn et al., 2001). Written informed consent was provided prior to the experiment and participants received course credit in return for their participation or took part on a voluntary basis.

### Experimental Design and Data Acquisition

**Fig. 1:**
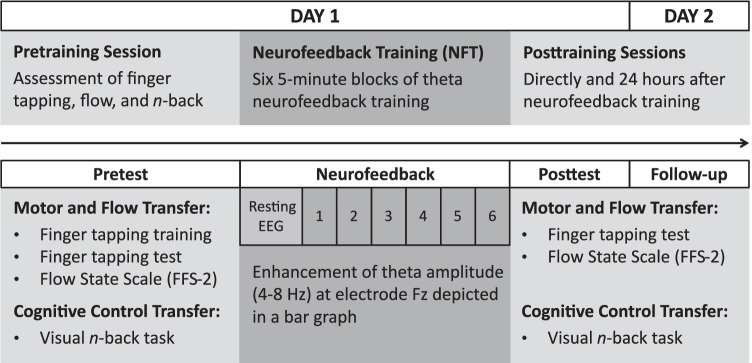
Overview of the neurofeedback training schedule. Neurofeedback consisted of fives minutes of resting EEG followed by six 5-minute training blocks. Training-induced enhancement in flow experience and performances in a finger tapping and an *n*-back task were investigated from one pretraining to two posttraining sessions

The training schedule consisted of three main stages, comprising a pre-training session, a 30-minute FM theta NFT session, and two posttraining sessions (Fig. 1). In the pre-training session, participants learned and practiced a finger tapping sequence before being tested on the number of correct repetitions. Afterwards, participants’ flow experience during the preceding finger tapping task was assessed with a German version of the Flow State Scale (FSS-2; Jackson et al., 2010). The FFS-2 was shown to reliably capture flow experience in motor tasks (Jackson et al., 2008). Furthermore, a visual *n*-back task was conducted in order assess training-induced changes in cognitive control processes. Measurement of flow experience was limited to the finger tapping task because the FSS-2 focuses on flow experience in physical activities. Moreover, another flow questionnaire capturing flow experience in the *n*-back task was not included in order to prevent interference between the different flow questionnaires. The subsequent NFT session consisted of six 5-minute blocks, during which participants trained to upregulate their FM theta amplitude. During the two posttraining sessions immediately and one day after NFT, finger tapping performance, flow experience, and *n*-back performance were assessed again. Assessment order of the finger tapping task including FSS-2 and the *n*-back task was counterbalanced across participants but kept constant over pre- and posttraining sessions. Notably, participants were made aware that a questionnaire, which asked about their experiences during finger tapping, followed task execution but flow experience was not mentioned specifically. Additionally, none of the statements in the FFS-2 included the word flow. Consequently, participants were not aware that the study aimed at investigating flow experience until the debriefing after the end of the study. For all sessions, participants were seated comfortably in a dimly lit and quiet experimental room and experimental stimuli and NFT were presented on a Dell Computer with a Dell 24-inch monitor placed at a viewing distance of approximately 70 cm.

#### Measurement of Motor Performance and Flow Experience

In order to explore the transfer of FM theta NFT to motor performance, a keyboard version of a finger tapping task (Rozengurt et al., 2016; Walker et al., 2005) was applied before, immediately, and 24 hours after NFT using E-Prime 2.0 software (Psychology Software Tools, Pittsburgh, USA). During pretest, participants trained to type the sequence 4-1-3-2-4 with their non-dominant left hand on a regular computer keyboard. The numbers 1-4 were marked on the keys “C”, “V”, “B”, and “N” and each finger was assigned to a different number with 1 = little finger, 2 = ring finger, 3 = middle finger, and 4 = index finger. Finger tapping training comprised ten blocks of 16 trials, during which a white fixation cross was displayed at the center of a black screen. Participants were instructed to correctly type the sequence once during each 2500-ms trial while fixating the fixation cross. The beginning of each trial was indicated by the last of three tones with a duration of 250 ms each. Training blocks were separated by 30-second breaks, during which a blank screen was presented.

After finger tapping training at pretest, participants’ motor performance was assessed with a finger tapping test that consisted of four 30-second trials. During these trials, participants were asked to continuously type the previously learned sequence as frequently and accurately as possible while fixating a white fixation cross that was centrally presented on a black background. Three 500-ms tones prepared participants for the beginning of a new trial and a fourth tone after 30 seconds served as a stop signal. In order to keep motivation high, participants received feedback about their performance after each trial and were asked to gradually increase their performance throughout each finger tapping test. Trials were separated by 50-second breaks including the display of the number of correctly typed sequences for 3000 ms and a subsequent blank screen. Motor performance for statistical analyses was defined as the number of correctly typed sequences during each participant’s best performance trial. The finger tapping test at posttest and follow-up measurement was administered without a preceding finger tapping training but participants were reminded of the correct sequence before the start of the test.

Flow state, that is, the specific subjective flow experience during finger tapping, was assessed with the long physical version of the FSS-2 (Jackson et al., 2010), which was administered directly after each finger tapping test. The original version of the FSS-2 was translated into German and evaluated by four independent consultants fluent in German and English, with one or the other being their mother tongue. The questionnaire consisted of 36 statements measuring nine flow dimensions with four statements each. The nine flow dimensions included three prerequisites for the occurrence of flow (challenge-skill balance, clear goals, unambiguous feedback) and six psychological aspects of flow experience (merging of action and awareness, concentration on the task at hand, sense of control, loss of self-consciousness, transformation of time, autotelic experience). Participants were instructed to judge each statement on a 5-point Likert scale with 1 = “strongly disagree” to 5 = “strongly agree”. Ratings of all statements were added up to a global flow score.

#### Measurement of *n*-back Task Performance

Transfer to cognitive control processes was measured by applying a visual *n*-back task (Schneiders et al., 2011) at pretest, posttest, and follow-up measurement using E-Prime 2.0 software (Psychology Software Tools, Pittsburgh, USA). The *n*-back task consisted of five 2-back blocks and five 0-back blocks that were presented in alternating order. During each block, 20 + *n* trials, which consisted of a white fixation cross for 2500 ms and a black-and-white pattern for 500 ms, were sequentially displayed against a gray background. Every block started with a 2300-ms display of “*n* = 2” or “*n* = 0” to indicate the following condition. Participants were asked to monitor the trial sequence and to respond to every displayed pattern. During 2-back blocks, participants were instructed to indicate whether the pattern was present two trials before or not. During 0-back blocks, they had to decide whether the pattern contained a gray dot at the center or not. Responses for “yes” and “no” were given by pressing the keys “D” and “L” with the left and right index finger, respectively. Response assignments were counterbalanced across participants but held constant across testing sessions. All blocks consisted of six target and 14 + *n* non-target trials and patterns were chosen from a sample of eight black-and-white patterns. Importantly, different patterns were chosen for the hit trials across blocks. A new sample of patterns was used for every testing session. Assignment of pattern samples to testing sessions was counterbalanced across participants. In order to familiarize participants with the task, the *n*-back task at each testing session was preceded by two practice blocks, one 2-back block with four trials, and one 0-back block with two trials. Accuracy that was measured by means of Pr scores (hits – false alarms) and reaction times of hits were assessed for statistical analyses.

#### NFT Protocol and Processing

For upregulation of FM theta activity during NFT, a self-built feedback protocol using ProComp5 Infinity amplifier and BioGraph Infinity software (Thought Technology Ltd., Montreal, Canada) was applied. The NFT protocol started with a 5-minute baseline measurement of resting-state EEG activity, during which participants had to fixate a white fixation cross on a black background. Throughout the following six 5-minute blocks of NFT, participants were instructed to raise a blue bar displaying their momentary FM theta amplitude as high and for as long as possible. Participants were given a list of strategies (e.g., mental imagery, relaxation, concentration) for increasing the feedback bar and were encouraged to find their own preferred strategy within the first two blocks. During the remaining four NFT blocks, participants were asked to constantly use the strategy that they believed to show the greatest benefit. Between blocks, participants were able to take self-paced breaks. To ensure that the same strategy was used in the last for blocks, participants were asked about the strategy they used in the preceding block. During NFT, electrophysiological activity was recorded with a 256-Hz sampling rate from an electrode placed at the Fz position (Eschmann et al., 2020; Rozengurt et al., 2016) that was referenced and grounded by two electrodes at the earlobes. Electrode impedances were kept below 5 kΩ. Frequency bands for feedback generation were extracted from raw EEG with an infinite impulse response (IIR) filter and amplitude changes were calculated as the root mean square (RMS) over a sliding window of 256 data points (equals one second) with a 300-ms butterworth buffer. Two frequency bands (0.5-2 Hz and 43-59 Hz) were extracted in order to detect eye and muscle activity. To assure a reliable elimination of artefacts, thresholds for the artefact frequency bands were set individually for each participant based on visual inspection. Whenever one or both of the thresholds were exceeded, the feedback bar turned red, indicating to the participant that there was an artifact. Immediately after NFT, a self-made questionnaire was applied that consisted of nine questions and asked participants about their current feelings (hunger, sleepiness, concentration, retentivity, joy, sadness) and experienced difficulty, motivation, and engagement during training. Responses were given on a 7-point Likert scale ranging from 1 = “very low” to 7 = “very high.”

Offline analyses of the NFT data was conducted with Brain Vision Analyzer 2.1 software (Brain Products GmbH, Gilching, Germany). Raw data of training and baseline blocks were filtered with a 0.1-40 Hz bandpass filter (48 dB/oct) and segmented into 1-second intervals. If segments contained artifacts indicated by a voltage step greater than ± 25 µV, they were discarded from further analysis. Frequency analysis was performed with a fast Fourier transformation with a 10% hamming window. Afterwards, results were averaged over all 1-second intervals and mean FM theta (4-8 Hz) and adjacent alpha (8-12 Hz) amplitudes were extracted for the baseline measurement and each NFT block. Alpha activity was extracted in addition to theta activity in order to investigate the specificity of the applied NFT intervention. If theta but not alpha activity increases based on NFT, training transfer can be ascribed specifically to the training-induced enhancement of theta activity (Gruzelier, 2014b).

### Data Analyses

#### NFT Effects

Training-induced FM theta change during NFT was calculated as relative increase of FM theta amplitudes from the baseline measurement to the respective training block. This procedure has the advantage that it controls for inter-individual differences in FM theta amplitudes. Overall, FM theta increase was investigated with a simple *t*-test that tested whether the mean FM theta increase of all blocks was different from 0. In order to investigate the specificity of FM theta NFT, the same analysis was conducted for alpha activity. If there was an overall activity change, increases or decreases across all training blocks were further explored with an one-way repeated-measures ANOVA with the within-subject factor Block (1-6).

**Fig. 2:**
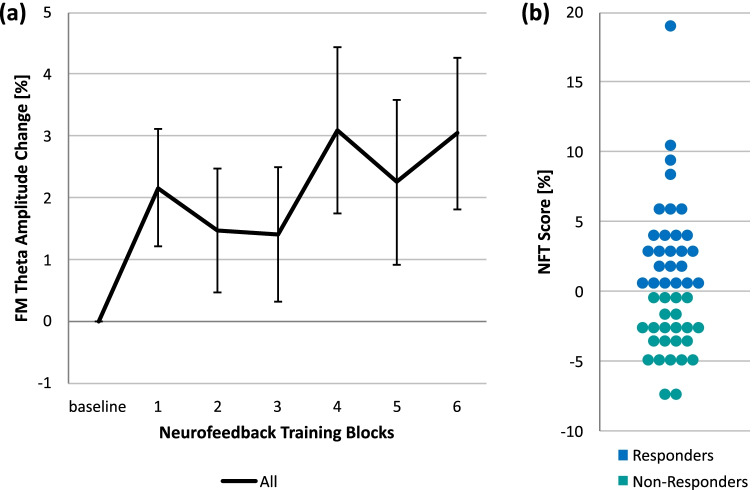
Neurofeedback training results over all participants and NFT scores for responders and non-responders. (**a)** Overall, participants were able to increase their FM theta activity relative to the baseline during NFT. Error bars indicate standard error of the mean. (**b)** Participants’ NFT score, that is, the percentage change of FM theta activity from the first two training blocks of different upregulation strategy usage to the remaining four training blocks of preferred strategy usage, was used to determine participants’ NFT success. Participants with a NFT score greater than zero were classified as responders, whereas participants with a NFT score smaller than zero were classified as non-responders. Thus, the NFT score reflects the ability to use the chosen strategy to increase FM theta activity successfully

In order to divide participants into responders and non-responders, an NFT score that reflected participants’ training success was calculated. This NFT score was defined as percentage change from the mean FM theta activity of the first two training blocks, during which participants were allowed to try different strategies in order to upregulate their brain activity, to the mean of the remaining four training blocks, during which they were asked to constantly use their preferred strategy (cf. Fig. 1). Previous studies, which also calculated NFT success as FM theta increase from phases of random strategy usage to phases of preferred strategy usage, found a relationship between the NFT score and training transfer to cognitive tasks (Eschmann & Mecklinger, 2021; Eschmann et al., 2020). These findings support the view that the NFT score reflects the successful choice and application of a strategy that allows for subsequent FM theta increase. In the present study, participants with an NFT score greater than zero were classified as responders whereas participants with an NFT score smaller than zero were categorized as non-responders (Fig. 2b). Of note, neurofeedback studies that also used a criterion of neurofeedback success greater/smaller than zero for classifying responders and non-responders resulted in equal sample sizes for responders and non-responders (Autenrieth et al., 2020; Hanslmayr et al., 2005). Given that the present study defined neurofeedback success differently and trained another frequency band, which restricts the comparability with the aforementioned studies, the present classification procedure resulted in approximately equal sample sizes rather coincidentally. Compared to neurofeedback studies that used the brain activity increase from baseline to neurofeedback as a classification criterion and found about one-third of participants to be non-responders (e.g., Enriquez-Geppert et al., 2014b; Zoefel et al., 2010), the stricter criterion used in the present study might make the applied neurofeedback seem less efficient. However, if there is an overall theta increase from baseline to neurofeedback in the present study, FM theta NFT is effective but a more fine-grained differentiation is necessary to compare successful with unsuccessful theta upregulators. In order to rule out that differences in baseline measurements caused differential FM theta increases for responders and non-responders, FM theta activity during baseline was compared between both groups with an independent samples *t*-test. Moreover, group differences in feelings and experienced difficulty, motivation, and engagement during training as assessed by a self-made questionnaire were assessed with additional *t*-tests.

#### Behavioral Transfer

General changes of motor performance, flow experience, and *n*-back performance from pre- to posttraining sessions were analyzed with separate one-way repeated-measures ANOVAs with the within-subject factor Session (pretest vs. posttest vs. follow-up). Differences between responders and non-responders at both posttraining sessions were investigated with separate multiple regression analyses that controlled for flow and performance differences between groups. If participants differ in their initial performance at pretest, they also differ in their possibility to profit from NFT and results of statistical analysis, which do not account for individual differences at pretest, might be biased. Therefore, flow or performance measures at pretest were included as a predictor of no further interest (cf. Eschmann et al., 2020). This procedure has the advantage of taking into account that participants with high initial flow experience and motor performance have less room for training-induced improvement compared to participants with low initial measures. Including pretest performance as a predictor variable into regression analyses controls for this variability in training-induced performance enhancement, and thus is essential for revealing genuine training transfer. For these analyses, regression coefficients *b* and *t*-tests solely for the predictor of interest, namely Group (responders = 1 vs. non-responders = 0), are reported. NFT effects on flow and performance increases were assessed with linear regressions with NFT score as predictor and enhancements from pre- to posttest as well as from posttest to follow-up as dependent variables. Flow and performance increases from pre- to posttest and from posttest to follow-up session were calculated as the percentage change between the respective measurements. For analyses of flow experience, additional multiple regressions were conducted that controlled for enhancement in motor performance and felt joy after training, which was measured after NFT with a self-made questionnaire, by including them as additional predictors. In addition to analyses of the global flow score, exploratory regression analyses were computed for all nine flow dimensions.

All statistical analyses were carried out with IBM SPSS Statistics 25 software (IBM Corp., New York, USA). For all analyses, the significance level was set to *α* = 0.05, and if not indicated differently two-tailed tests were used. Whenever necessary, Greenhouse-Geisser correction was applied and adjusted *p*-values are provided. In order to correct for multiple comparisons of post-hoc tests, the false discovery rate (FDR) method was applied and adjusted *p*-values, which were calculated using the p.adjust function in RStudio 1.2, are given (Benjamini & Hochberg, 1995). In order to avoid biases from outliers for all regression analyses, univariate outliers were detected with the Tukey method using three interquartile ranges (Tukey, 1977). Based on this outlier detection method, one non-responder was identified as an outlier for the flow enhancement from posttest to follow-up and removed from the respective regression analyses. Moreover, one responder was removed as an outlier from analysis of the flow dimension challenge-skill balance at the follow-up session. For accuracy analyses in the *n*-back task, one responder was an outlier in the 2-back condition at the follow-up session and consequently removed from all respective analyses. For reaction time analyses, one responder was an outlier in the 0-back condition at posttest and the follow-up session and another responder was an outlier in the 0-back condition at the follow-up session.

## Results

### NFT Effects

Overall, participants were able to enhance their FM theta activity during NFT above 0 (*M*_theta_ = 2.24%, *SE*_theta_ = 1.01%) as revealed by a significant *t*-test with the relative increase of FM theta activity from the baseline measurement to NFT blocks as dependent variable (*t*(47) = 2.21, *p* = .032, *d* = 0.32). As would be expected by the classification of participants into responders and non-responders based on FM theta NFT success, responders showed a theta increase of *M*_resp_ = 4.14% (*SE*_resp_ = 1.50%) and non-responders of *M*_non-resp_ = 0.16% (*SE*_non-resp_ = 1.23%). In contrast, alpha activity showed a non-significant decrease from baseline to neurofeedback (*M*_alpha_ =  - 3.08%, *SE*_alpha_ = 1.65%), which did not differ from 0 (*t*(47) = 1.87, *p* = .068, *d* = 0.27). Responders showed an alpha change of *M*_resp_ =  - 1.28% (*SE*_resp_ = 2.74%) and non-responders of *M*_non-resp_ =  - 5.03% (*SE*_non-resp_ = 1.68%). Overall changes in theta and alpha activity during NFT reliably differed from each other (*t*(47) = 3.46, p < .001, *d* = 0.50). An one-way repeated-measures ANOVA of baseline-corrected theta activity with the within-subject factor Block (1-6) was not significant (*F*(3.37,158.23), *p* = .263, *η*^2^ = .03), indicating that FM theta increase did not change across training blocks (Fig. 2a).

It could be argued that lower FM theta amplitudes during the baseline measurement allowed for greater FM theta increases and thus greater NFT scores of the responders (Fig. 2b; cf. Rozengurt et al., 2016). Comparison of baseline measurements between responders and non-responders with an independent samples *t*-test revealed no significant difference (*t*(46) = 0.07, *p* = .945, *d* = 0.02), indicating that both groups showed comparable FM theta amplitudes before training. Notably, analysis of the posttraining questionnaire revealed that both groups differed neither in their feelings after NFT nor in experienced difficulty, motivation, and engagement during training (all FDR-adjusted *p*-values > .266).

### Transfer to Motor Performance and Flow Experience

**Fig. 3:**
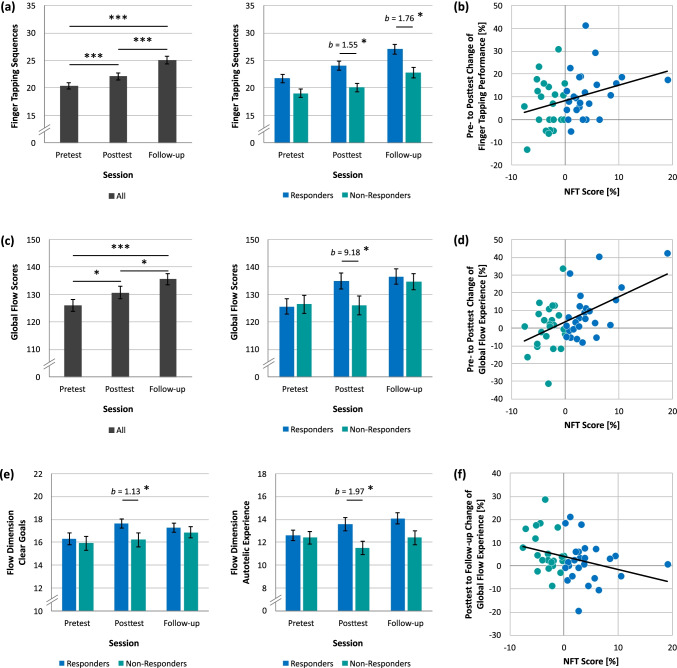
Training-induced changes in motor performance and flow experience. (**a)** Motor performance increased from pretest to posttest and to follow-up session for all participants as well as for responders relative to non-responders. (**b)** Participants’ NFT score, that is, the percentage change of FM theta activity from the first two training blocks to the remaining four training blocks, significantly predicted pre- to posttest enhancement of motor performance. (**c)** Global flow experience during finger tapping increased across all pre- and posttraining sessions but differed between responders and non-responders only at posttest. (**d)** The NFT score significantly predicted pre- to posttest flow enhancement−even if motor performance enhancement and joy felt after NFT was controlled for. (**e)** Responders indicated to have clearer goals and a greater autotelic experience compared to non-responders during finger tapping at posttest. (**f)** Participants with a smaller FM theta change during NFT showed a greater flow enhancement from posttest to follow-up session. However, this relationship was not significant when controlling for respective motor performance enhancement. Error bars indicate standard error of the overall and group means. * *p* < .05, ** *p* < .01, *** *p* < .001

Across all pre- and posttraining sessions, participants showed an increase in the number of correctly typed sequences in the finger tapping task as indicated by an one-way repeated-measures ANOVA with a significant main effect of Session (*F*(1.54,72.25) = 97.52, *p* < .001, *η*^2^ = .67; Fig. 3a). Significant post-hoc *t*-tests showed that the number of correctly typed sequences was higher at posttest as compared to pretest (*t*(47) = 5.83, *p* < .001, *d* = 0.84) as well as in the follow-up session relative to posttest (*t*(47) = 10.53, *p* < .001, *d* = 1.52) and pretest (*t*(47) = 11.11, *p* < .001, *d* = 1.60). As expected, responders were able to type more correct sequences after training than non-responders, which was revealed by a significant predictor of Group in the multiple regression analyses for posttest (*b* = 1.55, *t*(45) = 2.63, *p* = .012) and follow-up finger tapping performance (*b* = 1.76, *t*(45) = 2.04, *p* = .048). Linear regressions across all participants demonstrated that the NFT score was associated with the enhancement in finger tapping sequences from pretest to posttest (*b* = 0.67, *t*(46) = 2.23, *p* = .031), explaining 9.8% of the variance. This result indicates that the more successfully participants upregulated their FM theta activity during training, the larger was their increase in correctly typed sequences (Fig. 3b). In contrast, the NFT score was not linked to finger tapping enhancement from posttest to the follow-up session (*b* =  - 0.20, *t*(46) = 0.75, *p* = .456).

An one-way repeated-measures ANOVA revealed a significant main effect of Session (*F*(2,94) = 11.33, *p* < .001, *η*^2^ = .19), indicating that global flow experience during the finger tapping task increased across all pre- and posttraining sessions (Fig. 3c). In addition, significant FDR-corrected *t*-tests indicated that flow experience was higher at posttest as compared to pretest (*t*(47) = 2.05, *p* = .046, *d* = 0.30) as well as in the follow-up session relative to posttest (*t*(47) = 2.84, *p* = .010, *d* = 0.41) and pretest (*t*(47) = 4.80, *p* < .001, *d* = 0.69). As revealed by multiple regression analyses that controlled for flow measures at pretest, flow experience was higher for responders relative to non-responders at posttest (*b* = 9.18, *t*(45) = 2.34, *p* = .024) but not in the follow-up measurement (*b* = 2.37, *t*(45) = 0.69, *p* = .492), suggesting that non-responders experienced a similar level of flow as responders one day after NFT. Further multiple regressions for the nine flow dimensions revealed that responders had clearer goals (*b* = 1.13, *t*(45) = 2.88, *p* = .039) and a greater autotelic experience than non-responders at posttest (*b* = 1.97, *t*(45) = 2.75, *p* = .039; Fig. 3e), indicating that responders perceived finger tapping as more intrinsically rewarding compared to non-responders. Group differences for other flow dimensions at posttest or in the follow-up session were not significant (all FDR-adjusted *p*-values > .087). Linear regressions across all participants revealed that participants’ NFT score was associated with flow enhancement from pretest to posttest (*b* = 1.42, *t*(46) = 4.06, *p* < .001) and from posttest to follow-up measurement (*b* =  - 0.57, *t*(45) = 2.21, *p* = .033) explaining 26.3% and 9.8% of variance, respectively. While successful FM theta upregulation was associated with an increase in flow experience from pretest to posttest (Fig. 3d), this relationship reversed for the change of flow experience from posttest to follow-up measurement, where unsuccessful FM theta upregulation was associated with an increase flow experience (Fig. 3f). Participants with no flow enhancement from pretest to posttest showed an increase in flow experience from posttest to follow-up measurement and vice versa. On the one hand, if flow experience did not increase immediately after NFT due to unsuccessful FM theta upregulation, as it was the case for the non-responders, flow experience increased later after training possibly through posttraining consolidation processes of the learned motor movement. On the other hand, if flow already increased from pretest to posttest, as for responders in the present study, flow experience was not further enhanced from posttest to follow-up measurement, suggesting that these participants reached their maximum flow experience directly after FM theta upregulation. This fits to the finding that responders showed a greater level of flow experience compared non-responders at posttest but non-responders caught up to a similar level of flow experience as responders in the follow-up session (cf. Fig. 3c). Despite this finding, flow enhancement might be driven by enhancement in finger tapping performance and felt joy after NFT because responders may experience NFT as more rewarding than non-responders. Especially for non-responders, lower perceived joy after NFT might have had a detrimental effect on flow experience at posttest and posttraining consolidation processes occurring between posttest and follow-up measurement might have facilitated flow experience at the last posttraining session. Additional multiple regressions that controlled for enhancement in motor performance and felt joy after training showed that the NFT score was still associated with flow enhancement from pretest to posttest (*b* = 1.23, *t*(44) = 3.25, *p* = .002) but not from posttest to the follow-up session (*b* =  - 0.49, *t*(43) = 1.93, *p* = .060; Table 1). Nevertheless, motor performance enhancement was linked to flow enhancement from posttest to the follow-up session (*b* = 0.42, *t*(43) = 3.23, *p* = .002). These results suggest that pre- to posttest flow enhancement is driven by FM theta increase during training, whereas flow enhancement from posttest to the follow-up measurement is mainly influenced by enhancement in motor performance presumably based on posttraining consolidation processes.

**Table 1:** Multiple regressions of flow enhancement from pre- to posttest and from posttest to follow-up measurement while controlling for the respective motor performance enhancement and felt joy

Predictor	*B*	*SE*	*β*	*t*		*p*
Pre- to posttest flow enhancement						
Constant	0.07	7.81		0.01	.999	
Motor enhancement	0.25	0.18	.19	1.35	.185	
Joy after NFT	0.32	1.75	.03	0.18	.854	
NFT score	1.23	0.38	.44	3.25	.002**	
Posttest to follow-up flow enhancement						
Constant	- 2.36	5.60		0.42		.676
Motor enhancement	0.42	0.13	.42	3.23		.002**
Joy after NFT	0.18	1.10	.02	0.16		.874
NFT score	- 0.49	0.25	- .27	1.93		.060

*Note*. ^* ^*p* < .05, ** *p* < .01, *** *p* < .001

### Transfer to Cognitive Control

**Fig. 4:**
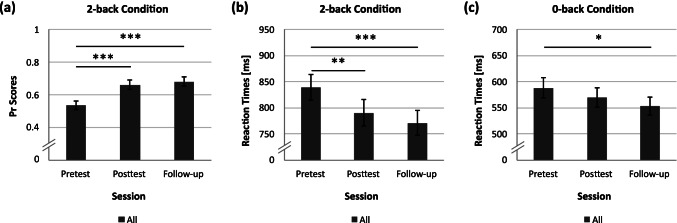
Transfer to cognitive control measured with a visual *n*-back task. (**a)** Pr scores in the 2-back condition increased from pretest to posttest but remained constant from posttest to the follow-up measurement. (**b)** Reaction times in the 2-back condition and (**c)** in the 0-back condition decreased from pretest to posttest but remained constant between posttest and follow-up measurement. There were no differences between responders and non-responders, indicating that performance enhancements are solely based on task repetition. * *p* < .05, ** *p* < .01, *** *p* < .001

Training-induced transfer to cognitive control was assessed with accuracy indicated by Pr scores and reaction times of hits in a visual *n*-back task. Performance and response speed in the 2-back condition increased across all pre- and posttraining sessions as shown by repeated-measures ANOVAs with significant main effects of Session for accuracy (*F*(2,92) = 23.22, *p* < .001, *η*^2^ = .34; Fig. 4a) and reaction times (*F*(2,94) = 11.96, p < .001, *η*^2^ = .20; Fig. 4b). Post hoc *t*-tests revealed that both accuracy and reaction times differed significantly between pretest and posttest (accuracy: *t*(46) = 5.01, *p* < .001, *d* = 0.73; reaction times: *t*(47) = 3.35, *p* = .002, *d* = 0.48) as well as between pretest and follow-up measurement (accuracy: *t*(46) = 6.35, *p* < .001, *d* = 0.93; reaction times: *t*(47) = 4.69, *p* < .001, *d* = 0.68), but there was no significant performance enhancement from posttest to the follow-up session (all FDR-adjusted *p*-values > .170). In the 0-back condition, reaction times decreased over all testing sessions (*F*(1.59,71.58) = 4.32, *p* = .024, *η*^2^ = .09; Fig. 4c), whereas accuracy remained stable (*F*(2,94) = 0.63, *p* = .537, *η*^2^ = .01). Reaction times in the 0-back condition differed only between pretest and follow-up measurement (*t*(45) = 2.57, *p* = .041, *d* = 0.38), while there was no difference between pretest and posttest (*t*(45) = 1.44, *p* = .156, *d* = 0.21) as well as between posttest and follow-up session (*t*(45) = 1.93, *p* = .091, *d* = 0.28). Multiple regressions that controlled for pretest performance indicated that there were no significant accuracy or reaction time differences between responders and non-responders neither at posttest nor in the follow-up session (all *p*-values > .095). Consequently, all observed performance increases in the *n*-back task can be ascribed to training-unspecific effects, such as task repetition, and not to FM theta enhancement by means of NFT.

## Discussion

The present study suggests that upregulation of FM theta activity via a 30-minute NFT session supports not only motor performance but also flow experience during a finger tapping task (Fig. 3a–d). Thereby, our results replicate and extend previous findings showing transfer of theta NFT to motor sequence learning (Reiner et al., 2014, 2018; Rozengurt et al., 2016). Given that motor enhancement lasted up to a week after training in previous studies, NFT in the present study might have a similar long-lasting effect on finger tapping performance in the present study. Specifically, individual differences in FM theta upregulation in the present study were associated with the increase of flow experience from pre- to posttest, even when corresponding increases in motor performance and feeling of joy after NFT were controlled for (Table 1). Notably, this relationship has to be interpreted with caution as it cannot be ruled out that individual differences in the ability to modify theta activity might play a crucial role in flow enhancement and not FM theta increase itself. Alternatively, this finding suggests that direct modulation of FM theta activity might foster the occurrence of flow states that are not driven by motor performance or training-induced emotions. This finding extends previous findings of heightened FM theta activity during flow states (Katahira et al., 2018) by suggesting that flow is associated with FM theta activity and can be supported with NFT. In contrast to the pretest to posttest flow increase, posttest to follow-up enhancement of flow experience was driven by posttraining motor enhancement (Table 1), suggesting that this enhancement was driven by consolidation processes taking place after training (Dudai et al., 2015). This might be because all participants received feedback about their FM theta activity during neurofeedback but only non-responders were not able to further upregulate it from the first two NFT blocks relative to the remaining four NFT blocks, during which one preferred strategy had to be used (Fig. 2). Consequently, in the present study, non-responders constitute a more conservative control group compared to active or passive control groups in other FM theta NFT studies (Enriquez-Geppert et al., 2014a; Rozengurt et al., 2016). Given that previous FM theta studies were able to show transfer to cognitive control processes (Enriquez-Geppert et al., 2014a; Eschmann & Mecklinger, 2021; Wang & Hsieh, 2013), it was assumed that upregulation of FM theta activity may support flow experience by altering cognitive control processes. However, one session of FM theta NFT did not induce transfer to the 2-back condition of a visual *n*-back task in the present study, indicating that either one NFT session is not sufficient or transfer to cognitive control tasks needs more time to unfold (Eschmann & Mecklinger, 2021). If transfer to cognitive control processes, which emerges late after repetitive application of several NFT sessions, mediates training-induced flow enhancement, differences in flow experience between responders and non-responders might re-emerge late after repetitive NFT.

Based on the findings of the present study, two mechanisms of how FM theta NFT might support the occurrence of flow experience are conceivable. First, training-induced increases of FM theta activity might have led to enhancement of motor performance and associated flow experience by promoting consolidation of the learned motor movement (Reiner et al., 2014, 2018; Rozengurt et al., 2016). In turn, this enhanced consolidation might have diminished cognitive control demands after training, allowing increased flow experience to occur. In support of this, flow states were previously shown to be accompanied by a reduction in ACC activation that was interpreted as reflecting a lowered need for cognitive control (Klasen et al., 2012; Ulrich et al., 2016). Due to the immediacy of transfer effects right after NFT, it seems likely that FM theta upregulation promoted immediate consolidation of motor memory via synaptic consolidation (Dudai et al., 2015; Rozengurt et al., 2016). Recurrent synaptic consolidation has been suggested to support systems consolidation that transfers hippocampus-dependent memories into neocortical structures over time (Dudai, 2012; Frankland & Bontempi, 2005). Notably, theta oscillations seem to play a crucial role in the reactivation and subsequent consolidation of memory representations by allowing information transfer between hippocampal and PFC regions via theta phase synchronization (Benchenane et al., 2010). In the present study, FM theta upregulation might have supported the reactivation and consolidation of the learned motor memory representations by enhancing recurrent firing of neurons that were active during learning of the motor movement. Additionally, theta oscillations also could have supported motor consolidation by sharpening memory representations that compete for retrieval. It has been suggested that theta oscillations reflect varying levels of inhibition strength that lead to strengthening of target memories while competing memories are being suppressed (Norman et al., 2005, 2006). In the present study, memory representations of the correctly typed finger tapping sequence might have competed with motor representations of falsely typed sequences. FM theta upregulation might have facilitated the differentiation of the correct motor movement from interfering representations, leading to better motor performance after training. Irrespective of the mechanism by which FM theta NFT supported consolidation of motor memory representations, increased flow experience might have been induced by diminished cognitive control demands based on training-induced consolidation. Given that responders showed a higher increase of theta activity during neurofeedback than non-responders, subsequent memory consolidation should have been more pronounced, leading to a greater reduction of cognitive control demands. This interpretation would be in line with the definition of flow as a feeling of automatic and effortless control (Csíkszentmihályi, 1997) and findings of reduced ACC activation as a signal of tempered need for cognitive control during flow states (Klasen et al., 2012; Ulrich et al., 2016). Furthermore, FM theta decreases after NFT, possibly reflecting reduced control demands, have been associated with increased memory control task performance (Eschmann et al., 2020).

Second, another explanation for training-induced enhancements of motor performance and flow experience is that upregulation of FM theta activity supported cognitive control processes, which consequently benefited task goal maintenance and coordinated motor memory retrieval more efficiently, helping the feeling of flow during finger tapping to emerge. Although the present study did not reveal training transfer to a visual *n*-back task, previous studies that conducted more than one FM theta NFT session demonstrated transfer to cognitive and memory control processes (Enriquez-Geppert et al., 2014a; Eschmann & Mecklinger, 2021; Eschmann et al., 2020; Wang & Hsieh, 2013). More specifically, transfer was found in tasks, during which one stimulus or a temporal sequence of several stimuli had to be actively maintained in working memory (Enriquez-Geppert et al., 2014a; Eschmann & Mecklinger, 2021). In line with these findings, theta oscillations have been suggested to be especially beneficial for the maintenance and retrieval of sequential order memory (e.g., Heusser et al., 2016; Hsieh et al., 2011; Lisman & Jensen, 2013; Roberts et al., 2014). Consequently, FM theta upregulation in the present study may have supported the reinstatement and active maintenance of the learned finger tapping sequence, thereby reflecting enhanced task goal maintenance that leads to better motor performance after training. Support for this interpretation comes from studies demonstrating heightened lateral PFC activation during conditions of flow experience, which were assumed to reflect increased cognitive control processes, such as task goal maintenance and top-down control (Ulrich et al., 2014, 2016; Yoshida et al., 2014). Moreover, a substantial level of upregulation and increased connectivity between brain regions involved in cognitive control and reward processing seems to be crucial for the experience of flow (Huskey et al., 2018a, 2018b; Klasen et al., 2012). With regard to electrophysiological measures, FM theta activity as an indicator of cognitive control was shown to be enhanced during flow experience (Takahashi et al., 2005). In the present study, responders reported higher ratings in the global flow dimensions of clear goals and autotelic experience compared to non-responders after NFT training. While greater autotelic experience reflects stronger feelings of joy and satisfaction during task completion, clearer goals might mirror increased task goal maintenance. Similarly, mindfulness meditation interventions that have been associated with cognitive control processes (Tang et al., 2015) have been shown to enhance scores on the clear goals and sense of control flow dimensions (Aherne et al., 2011), suggesting that both mindfulness and neurofeedback interventions result in comparable flow enhancements. Taken together, the two explanations for an enhanced flow experience based on diminished control demands and increased cognitive control processes might seem contradictory but are not necessarily mutually exclusive. In addition to differential findings of activation increases and decreases in control-related brain areas during flow experience (Klasen et al., 2012; Ulrich et al., 2014, 2016; Yoshida et al., 2014), it has been shown that even though flow states are subjectively perceived as effortless, they involve effortful, objectively measurable control processes (Harris et al., 2017a) and activation of and connectivity between control-related and reward-associated brain areas (Huskey et al., 2018a, 2018b; Klasen et al., 2012). Hence, flow-eliciting situations seem to be characterized by a dichotomous relationship between perceived control demands and utilization of cognitive control processes, which both might have been altered by FM theta NFT in the present study.

A limitation of the present study is that the relationship between participants’ NFT score and enhancement of motor performance and flow experience is correlational in nature. Given that participants were split into responders and non-responders based on their NFT success, subsequent enhancement of motor performance and flow experience might not be induced solely by FM theta increase. It is conceivable that participants who are better able to increase their theta activity, such as the responders in the present study, are also better in enhancing their motor performance and flow experience across pre- and posttraining sessions. Consequently, the ability to both increase FM theta activity and enhance performance per se might play a crucial role in the discovered relationships. Even though other NFT studies have also investigated the impact of NFT on performance by contrasting responders and non-responders (e.g., Autenrieth et al., 2020; Hanslmayr et al., 2005), studies with active control groups, who train the upregulation of other frequency bands (e.g., Eschmann et al., 2020; Rozengurt et al., 2016), are required to rule out such alternative explanations. Thus, the present findings are preliminary and should be interpreted with caution. The multiple regression analyses in the present study can at least rule out the influence of felt joy after NFT and the ability to enhance motor performance by controlling for both.

If studies with active control groups support the idea that FM theta increase induces flow experience during motor movements, FM theta NFT may constitute an alternative to mindfulness mediation interventions that have been used for flow enhancement in the field of competitive sports thus far (Gardner & Moore, 2012; Kaufman et al., 2009). Comparing mindfulness meditation with NFT, it seems reasonable to assume that both interventions rely on similar cognitive control processes needed for successful self-regulation (Hofmann et al., 2012). Both training techniques involve continuous self-monitoring of one’s own inner state as well as the administration of top-down control in order to change this state in the desired direction and maintain the altered outcome (e.g., Gaume et al., 2016; Teper et al., 2013). In support of this assumption, two key regions of the cognitive control network, namely the ACC and PFC, were shown to be crucial for mindfulness meditation and NFT (e.g., Hölzel et al., 2007; Ninaus et al., 2013; Sitaram et al., 2017; Tang et al., 2015). Interestingly, the morphology of the ACC, a brain region important for conflict detection, was related to NFT success with smaller ACC volumetry associated with non-responsiveness to neurofeedback (Enriquez-Geppert et al., 2013; Ninaus et al., 2015). Additionally, the proneness to experience flow has been linked to individual differences in dopaminergic function in the dorsal striatum, a brain region which is important for reward processing and intrinsic motivation (de Manzano et al., 2013). In consequence, individual differences in the neurophysiological underpinnings of neurofeedback learning and flow experience determine the success of a flow-promoting NFT intervention and must therefore be taken into account. This suggestion receives further support from a study showing that flow-inducing situations, which are characterized by a challenge-skill balance, are associated with increased functional connectivity between key regions of cognitive control and reward networks, indicating that flow as a form of intrinsic reward contributes to cognitive control allocation (Huskey et al., 2018a). In contrast to mindfulness interventions, various NFT protocols have been used in order to enhance athletic performance, but resulted in mixed outcomes (Gruzelier, 2013, 2014a; Jeunet et al., 2019; Mirifar et al., 2017). For instance, upregulation of sensory motor rhythms at central electrode sites resulted in better golf putting performance of the training relative to a control group (Cheng et al., 2015), whereas combined downregulation of alpha and theta activity at frontal-midline sites failed to show transfer to golf putting performance (Ring et al., 2015). Moreover, it remains unclear whether training-induced motor enhancements in these studies were also accompanied by increased experience of flow due to a decrease in control demands or increased use of cognitive control processes. Given that FM theta oscillations reflect a general mechanism for cognitive control (Cavanagh & Frank, 2014), upregulation of FM theta activity might be beneficial for a wide range of motor movements and thus sport disciplines. In the present study, a 30-minute NFT session also resulted in flow enhancement that was unrelated to increase in motor performance. Based on the immediacy of this transfer effect, it seems advisable to apply FM theta NFT directly before athletic training or competition. Similarly to a NFT study that applied real-life neurofeedback during golf putting (Arns et al., 2007), FM theta NFT might also be combined with the motor movement that is aimed to be improved if the sport discipline allows for an artifact-free EEG measurement. Compared to often long-lasting mindfulness interventions (Tang et al., 2010), NFT has the advantage that it can induce effects even with a short training session and that it involves direct feedback about one’s brain activity, supporting users to learn effective self-regulation quickly. Repetitive NFT application might reveal whether FM theta activity has also long-lasting effects on flow experience.

Altogether, the present study is among the first to suggest that FM theta NFT supports flow experience in newly learned motor movements. Importantly, flow improvements directly after training were unrelated to corresponding motor enhancements and felt joy after NFT, indicating a relationship between FM theta upregulation and increased flow experience. Consequently, FM theta NFT might constitute a promising tool for the induction of flow in competitive settings, such as amateur and elite sports.

## Declarations

### Open Practices Statement

The data, on which this paper is based, and the analysis scripts are available at https://osf.io/k3znw/.
